# Computational Imaging: The Next Revolution for Biophotonics and Biomedicine

**DOI:** 10.3390/cells13050433

**Published:** 2024-02-29

**Authors:** An Pan, Baoli Yao, Chao Zuo, Fei Liu, Jiamiao Yang, Liangcai Cao

**Affiliations:** 1 State Key Laboratory of Transient Optics and Photonics, Xi’an Institute of Optics and Precision Mechanics, Chinese Academy Sciences, Xi’an 710119, China; yaobl@opt.ac.cn; 2 Smart Computational Imaging Laboratory (SCILab), Department of Electronic and Optical Engineering, Nanjing University of Science and Technology, Nanjing 210094, China; zuochao@njust.edu.cn; 3 School of Optoelectronic Engineering, Xidian University, Xi’an 710071, China; feiliu@xidian.edu.cn; 4 Department of Instrument Science and Engineering, Shanghai Jiao Tong University, Shanghai 200240, China; jiamiaoyang@sjtu.edu.cn; 5 Department of Precision Instruments, Tsinghua University, Beijing 100084, China; clc@tsinghua.edu.cn

This Editorial is the preface for the topical collection of “Computational Imaging for Biophotonics and Biomedicine”, which collates the 12 contributions listed in Table 1. Computational imaging is the joint design of front-end optics and post-detection signal processing. In some cases, measurements made via this imaging technique may not even resemble a commonsense image. The raw data are sometimes regarded as coded, and the corresponding digital decoded processing is usually required to extract an expected image. Using the principles of computational imaging, one can design an instrument with reduced requirements in terms of size, weight, power, or cost to perform optical measurements with a capacity exceeding the physical limits of optics.

A key feature of computational imaging that distinguishes it from its conventional optical counterpart is feedback-based optimization to perform paired encoding and decoding methods. The post-processing of computational imaging is no longer a common method for most scenes. Another key feature of computational imaging is that it can perform multimodal imaging, that is, the fusion of a number of different imaging techniques. For example, imaging techniques such as bright-field, dark-field, phase, polarization, spectral, light-field, fluorescence, and volume can be used simultaneously to provide more comprehensive and accurate information. In addition, computational imaging can also reconstruct and enhance the image, improving its resolution and contrast, making details hidden in the noise, and making the background more visible.

One key trend in computational imaging is its integration with emerging technologies such as artificial intelligence and machine learning. These technologies can be used to automate image-processing tasks, enhance image quality, achieve digital staining, and enable new applications in fields such as medical imaging, remote sensing, and microscopy.

The other trend is the development of advanced computational techniques for image reconstruction and analysis; these enable researchers to extract more information from optical images simultaneously, such as depth information, surface topography, and material properties, by processing raw data in diverse ways.

Overall, the technological trend in computational imaging is directed towards more sophisticated and intelligent algorithms that enable researchers to push the boundaries of what is possible with optical imaging techniques. This has the potential to permeate a wide range of fields and lead to exciting new applications and discoveries.

From the perspective of applications, computational imaging may represent the next revolution in biophotonics and biomedicine. It integrates knowledge from the fields of computer science, optics, and biomedicine, and by utilizing advanced computational algorithms and image processing techniques, it allows us to observe and analyze the structure and function inside living organisms, tumors, and cells in unprecedented ways. While traditional imaging techniques often only provide simple static images or videos, computational imaging can provide more detailed and comprehensive information. It can extract useful biological information from complex optical signals by processing and analyzing large amounts of data. This technology has a wide range of applications in the biomedical field, e.g., helping doctors more accurately diagnose diseases, monitor the effects of treatments, and study biological processes.

Computational imaging also holds great potential for non-invasive imaging in living organisms, i.e., without the need for surgery or the insertion of instruments. This is important for research and diagnosis because it reduces harm and discomfort to patients.

Overall, computational imaging would be an important revolution in biophotonics and biomedicine. It will bring new breakthroughs in biomedical research and clinical diagnosis by integrating computer science and optical technologies to provide more comprehensive, accurate, and non-invasive imaging.

## Figures and Tables

**Table 1:** Overview of publications that are assembled in the topical collection.

Number	Type	Field	Title
1	Research article	Deep learning, data analysis.	A Novel Attention-Mechanism Based Cox Survival Model by Exploiting Pan-Cancer Empirical Genomic Information
2	Research article	Optical microscopy, Fourier ptychographic microscopy.	Fourier Ptychographic Microscopy via Alternating Direction Method of Multipliers
3	Research article	Deep learning, segmentation.	Deep Learning Based Real-Time Semantic Segmentation of Cerebral Vessels and Cranial Nerves in Microvascular Decompression Scenes
4	Research article	Optical microscopy, on-chip microscopy.	Pixel Super-Resolution Phase Retrieval for Lensless On-Chip Microscopy via Accelerated Wirtinger Flow
5	Research article	Deep learning, recognition.	A Deep-Learning Based System for Rapid Genus Identification of Pathogens under Hyperspectral Microscopic Images
6	Research article	Optical microscopy, transformer networks.	ContransGAN: Convolutional Neural Network Coupling Global Swin-Transformer Network for High-Resolution Quantitative Phase Imaging with Unpaired Data
7	Research article	Pseudo-F statistics, recognition.	HSSG: Identification of Cancer Subtypes Based on Heterogeneity Score of A Single Gene
8	Review	Optical microscopy, portable microscopy.	Computational Portable Microscopes for Point-of-Care-Test and Tele-Diagnosis
9	Research article	Deep learning, recognition.	Rapid Identification of Infectious Pathogens at the Single-Cell Level via Combining Hyperspectral Microscopic Images and Deep Learning
10	Research article	Machine learning, assessment.	Assessment of Primary Human Liver Cancer Cells by Artificial Intelligence-Assisted Raman Spectroscopy
11	Research article	Deep learning, segmentation.	FastCellpose: A Fast and Accurate Deep-Learning Framework for Segmentation of All Glomeruli in Mouse Whole-Kidney Microscopic Optical Images
12	Review	Optical microscopy, Fourier ptychographic microscopy.	Fourier Ptychographic Microscopy 10 Years on: A Review

## Data Availability

Not applicable.

